# Using Peace Corps volunteers in community eye health

**Published:** 2009-06

**Authors:** Shawn Barnes

**Affiliations:** Co-founder, Outbound Eye Health International, John A. Burns School of Medicine, University of Hawaii, Kakaako, Hawaii 96813, USA

Initiating a community eye health programme in an unfamiliar culture and language can be a daunting task. This report focuses on an underutilised resource for community eye health: American Peace Corps volunteers.

The Peace Corps is a government-sponsored service organisation. At present, there are 7,876 Peace Corps volunteers serving in 76 low- and middle-income countries around the world. Of those who volunteer, 94 per cent hold at least an undergraduate degree and 21 per cent are specifically trained to work in public health.^1^ Many volunteers serve in rural areas and all receive two months of intensive language and cultural training in their host countries; they also live at the financial level of those they serve. This represents a significant resource of educated, culturally and linguistically competent, and generally idealistic people who are willing to assist in community health projects. All one need do is ask for their assistance!

## Linguistic competence

Few eye care professionals have reported making use of this vast resource. However, those who have done so have commented on the logistical, organisational, and linguistic assistance of Peace Corps volunteers in setting up and carrying out eye health programmes in places like Fiji^2^ and Costa Rica.^3^ The cultural and linguistic competence of these volunteers can be of great assistance in community outreach and education.

In July 2009, I co-organised a project on the rural island of Savaii, Samoa, which provided full eye examinations for over 1,100 people. Screening was performed in 12 different villages around the island with no national government assistance. All logistics, liaison, and communication with villages were done by current or former Peace Corps volunteers. This provided an immense advantage as villagers sometimes view national government agencies with suspicion. By contrast, Peace Corps volunteers tend to be viewed as neutral and are generally not seen as any threat to village independence or land ownership.

**Figure:**
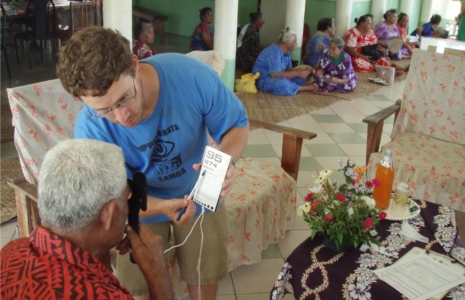
A Peace Corps volunteer performs a basic eye exam. SAMOA

Shawn Barnes

Therefore, the project was seen to be separate from national government and we were enthusiastically welcomed wherever we went.

In addition to logistical assistance, Peace Corps volunteers became the backbone of the eye examination team. After training in basic examination techniques such as near/far visual acuity, autorefraction, and use of the tonopen, Peace Corps volunteers were able to assist as technicians in eye examinations. They were also able to explain, in the Samoan language, what the team was doing and why.

Usha Raman recently pointed out^4^ that a bottom-up approach to eye care human resources should be emphasised in order to minimise the importance of the single ophthalmologist and maximise the human resource potential of the larger community in need.^4^ As long-term residents of these communities, and with linguistic and cultural competency, Peace Corps volunteers may serve as ideal intermediaries to help build a bridge between outside eye care professionals and the communities they wish to serve, as well as establishing connections to national health services. In addition, participation in a community service project is well in line with the mission of the Peace Corps. I would encourage any eye care professionals planning to work in low- and middle-income countries to find out whether there are Peace Corps volunteers serving in the area and make contact. Most Peace Corps volunteers will be glad to help.

To find Peace Corps volunteers, contact the Peace Corps office in Washington DC (*www.peacecorps.gov*) and ask to be connected with your local Peace Corps country director.
